# Anti-inflammatory effects of *Perilla frutescens* in activated human neutrophils through two independent pathways: Src family kinases and Calcium

**DOI:** 10.1038/srep18204

**Published:** 2015-12-14

**Authors:** Chun-Yu Chen, Yann-Lii Leu, Yu Fang, Chwan-Fwu Lin, Liang-Mou Kuo, Wei-Che Sung, Yung-Fong Tsai, Pei-Jen Chung, Ming-Chung Lee, Yu-Ting Kuo, Hsuan-Wu Yang, Tsong-Long Hwang

**Affiliations:** 1 Graduate Institute of Natural Products, School of Traditional Chinese Medicine, College of Medicine, Chang Gung University, Taoyuan 333, Taiwan; 2 Graduate Institute of Clinical Medical Sciences, College of Medicine, Chang Gung University, Taoyuan 333, Taiwan; 3 Department of Anesthesiology, Chang Gung Memorial Hospital, Taoyuan, Taiwan; 4 Department of Cosmetic Science, Chang Gung University of Science and Technology, Taoyuan 333, Taiwan; 5 Division of General Surgery, Department of Surgery, Chang Gung Memorial Hospital, Chiayi, Taiwan; 6 Brion Research Institute of Taiwan, New Taipei City 231, Taiwan; 7 Chinese Herbal Medicine Research Team, Healthy Aging Research Center, Chang Gung University, Kweishan, Taoyuan 333, Taiwan; 8 Department of Cosmetic Science and Research Center for Industry of Human Ecology, Chang Gung University of Science and Technology, Taoyuan 333, Taiwan

## Abstract

The leaves of *Perilla frutescens* (L.) Britt. have been traditionally used as an herbal medicine in East Asian countries to treat a variety diseases. In this present study, we investigated the inhibitory effects of *P. frutescens* extract (PFE) on N-formyl-Met-Leu-Phe (fMLF)-stimulated human neutrophils and the underlying mechanisms. PFE (1, 3, and 10 μg/ml) inhibited superoxide anion production, elastase release, reactive oxygen species formation, CD11b expression, and cell migration in fMLF-activated human neutrophils in dose-dependent manners. PFE inhibited fMLF-induced phosphorylation of the Src family kinases (SFKs), Src (Tyr416) and Lyn (Tyr396), and reduced their enzymatic activities. Both PFE and PP2 (a selective inhibitor of SFKs) reduced the phosphorylation of Burton’s tyrosine kinases (Tyr223) and Vav (Tyr174) in fMLF-activated human neutrophils. Additionally, PFE decreased intracellular Ca^2+^ levels ([Ca^2+^]_i_), whereas PP2 prolonged the time required for [Ca^2+^]_i_ to return to its basal level. Our findings indicated that PFE effectively regulated the inflammatory activities of fMLF-activated human neutrophils. The anti-inflammatory effects of PFE on activated human neutrophils were mediated through two independent signaling pathways involving SFKs (Src and Lyn) and mobilization of intracellular Ca^2+^.

*Perilla frutescens* (L.) belongs to the Lamiaceae family and is widely used as a common vegetable crop, condiment, and traditional herbal medicine in East Asian countries^1^,^2^. In traditional medicine, the leaves of *P. frutescens* are utilized to treat various illnesses such as cough, respiratory tract infection, food poisoning, diarrhea, and premature delivery^3^,^4^. The compounds contained in *P. frutescens* have attracted interest for their biological activities, which include anti-inflammatory^5^,^6^, anti-oxidative^7^,^8^, anti-HIV^9^,^10^, anti-tumor^11^, and anti-microbial functions^12^. Studies also indicated that *P. frutescens* can decrease inflammatory responses in immune cells, such as macrophages^13^,^14^ and mast cells^2^,^15^. However, the biological effects of *P. frutescens* on human neutrophils and the molecular mechanisms underlying these remain poorly understood.

Neutrophils are the most plentiful leukocyte in human blood, accounting for about 50-75% of circulating leukocytes. During inflammation, they are the first immune cells to arrive and execute their pathogen-eliminating function via multiple intra- and extracellular mechanisms^16^,^17^,^18^. However, the reactive oxygen species (ROS) and lytic enzymes can also damage healthy surrounding tissue, resulting in deleterious inflammatory diseases, such as acute lung injury, chronic obstructive pulmonary disease, and asthma^19^,^20^,^21^,^22^. In order to ameliorate these conditions, many studies have investigated the pharmacological modulation of activated human neutrophils by natural products and their mechanisms of action.

This present study investigated the modulatory effects of a *P. frutescens* var. *crispa* extract (PFE) in activated human neutrophils. We found that a non-toxic level of PFE reduced superoxide anion (O_2_˙^–^) production, elastase release, ROS formation, CD11b expression, and chemotactic migration in N-formyl-Met-Leu-Phe (fMLF)-induced human neutrophils. Neutrophils express the formyl-peptide receptor (FPR) that sense invading pathogen and tissue damage. Diverse intracellular signaling pathways, including G-proteins, calcium (Ca^2+^) mobilization , tyrosine protein kinases, adapter proteins, and cytoskeletal rearrangement are triggered by FPR and are responsible for neutrophil activation^23^. Many of the observations made in this study demonstrated that the anti-inflammatory effects of PFE were mediated through two pathways: blockade of Src family kinases (SFKs) and reducing intracellular Ca^2+^ mobilization.

## Results

### PFE inhibited O_2_˙^−^ production, elastase release, and ROS formation in fMLF-activated human neutrophils

**Figure 1:**
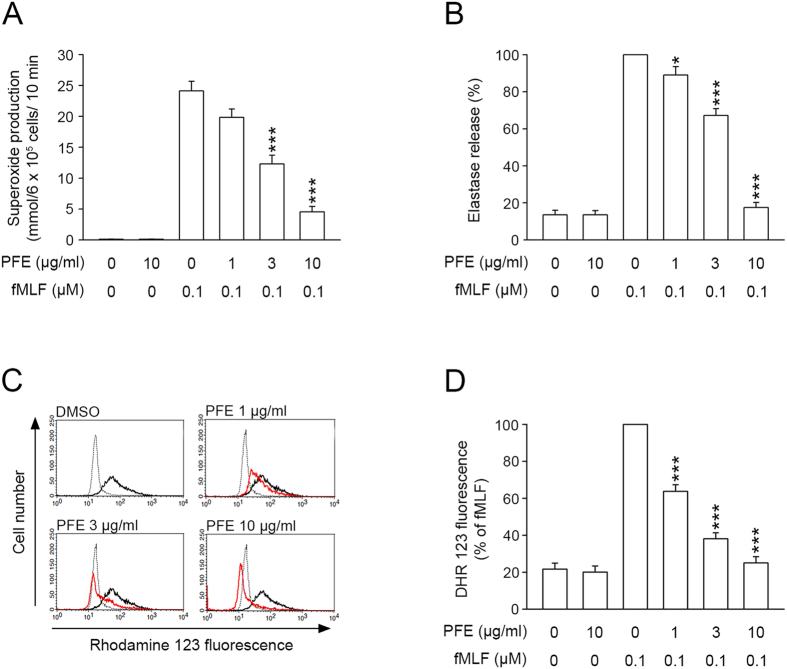
*P. frutescens* extract (PFE) inhibited O_2_˙^–^ production, elastase release, and formation of reactive oxygen species (ROS) in N-formyl-Met-Leu-Phe (fMLF)-activated human neutrophils. Human neutrophils (6 × 10^5^ cells/ml)were pre-incubated with dimethylsulfoxide (DMSO) or PFE (1, 3, and 10 μg/ml) and then activated with fMLF (0.1 μM). (**A,B**) O_2_˙^–^ and elastase release were detected spectrophotometrically using cytochrome *c* reduction and elastase substrate, respectively. (**C**) The fluorescence intensity of dihydrorhodamine 123 (DHR123) was used to detect the intracellular ROS. The ROS formation in fMLF-activated neutrophils pretreated with PFE (red line) was decreased, as compared with those without PFE (black line). The dashed line indicated neutrophils that were not treated with fMLF (basal group). (**D**) The mean values of fluorescence intensity from panel C. Data are expressed as the mean ± standard error of the mean, n = 7, *p < 0.05, ***p < 0.001, as compared to the fMLF group.

In order to evaluate whether PFE affected neutrophil function and inflammatory responses, we first investigated the effects of PFE on O_2_˙^−^ production, elastase release, and ROS formation in fMLF-activated human neutrophils. Our experiments revealed that O_2_˙^–^ and elastase, which were detected by ferricytochrome *c* and elastase substrate, respectively, were reduced by PFE (1, 3, and 10 μg/ml) in a concentration-dependent manner, with IC_50_ values of 3.18 ± 0.32 and 3.82 ± 0.27 μg/ml, respectively (Fig. 1A,B). ROS formation in activated neutrophils was also inhibited by PFE, with an IC_50_ value of 1.93 ± 0.24 μg/ml (Fig. 1C,D). PFE (10 μg/ml) did not affect basal O_2_˙^–^ production or elastase release in unstimulated neutrophils (Fig. 1A,B). Furthermore, our experiments showed that PFE (10 μg/ml) did not promote lactate dehydrogenase (LDH) release (data not shown), suggesting that PFE did not exert cytotoxic effects in human neutrophils.

### PFE inhibited CD11b expression and cell migration in fMLF-activated human neutrophils

**Figure 2:**
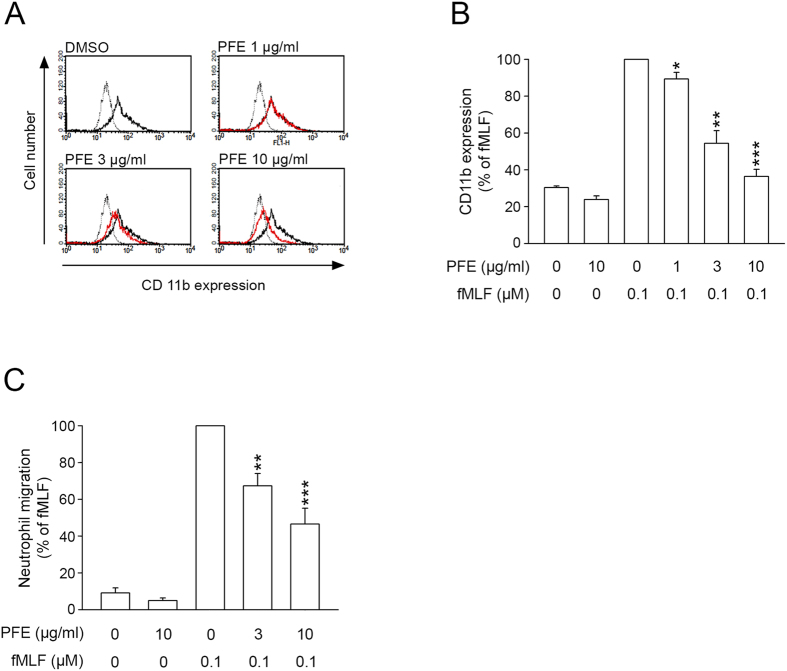
*P. frutescens* extract (PFE) inhibited CD11b expression and cell migration in N-formyl-Met-Leu-Phe(fMLF)-activated human neutrophils. Human neutrophils (5 × 10^6^ cells/ml) were pre-incubated with dimethylsulfoxide (DMSO) or PFE (1, 3, and 10 μg/ml) for 5 min and then activated with fMLF/cytochalasin B (CB) for another 5 min. (**A**) The fluorescence intensity of FITC-labeled anti-CD11b was monitored using flow cytometry in fMLF-activated neutrophils pretreated with PFE (red line), not pretreated with PFE (black line), and treated with DMSO (basal group, dashed line). (**B**) The mean values of fluorescence intensity from panel A. (**C**) Neutrophils (5 × 10^6^ cells/ml) were pre-incubated with DMSO or PFE (1, 3, and 10 μg/ml) for 5 min in the top chamber. Migrated neutrophils were counted after 60 min. Data are expressed as the mean ± standard error of the mean, n = 3–4, *p < 0.05, **p < 0.01, ***p < 0.001, as compared to the fMLF group.

CD11b/CD18 is involved in cellular adhesion between activated neutrophils and endothelial cells. When neutrophils are stimulated, they rapidly immobilize through activation of integrin CD11b/CD18, and subsequent modulation of this attachment allows migration. Our results demonstrated that fMLF stimulation of human neutrophils resulted in significant up-regulation of CD11b expression, while PFE (1, 3, and 10 μg/ml) inhibited CD11b expression in fMLF-activated human neutrophils with an IC_50_ value of 4.49 ± 1.39 μg/ml (Fig. 2A,B). Furthermore, fMLF-induced neutrophil migration was reduced in the presence of PFE (3 and 10 μg/ml; Fig. 2C). The IC_50_ for this effect was 5.36 ± 1.06 μg/ml.

### PFE inhibited the activation of SFKs

**Figure 3:**
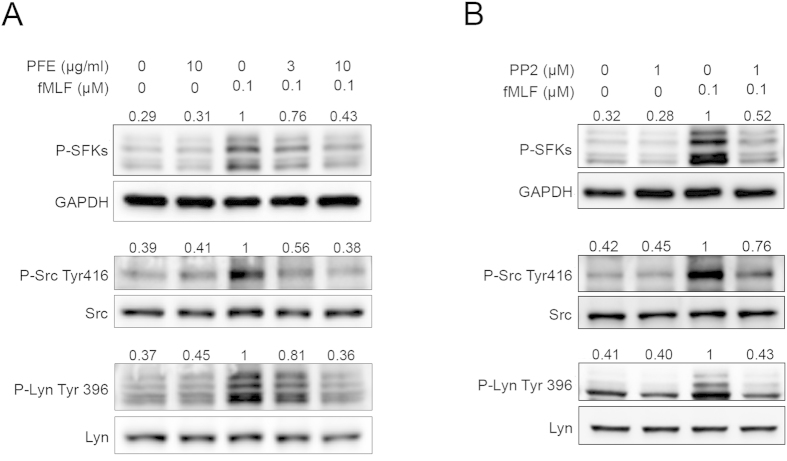
*P. frutescens* extract (PFE) inhibited the phosphorylation of Src family kinases (SFKs) in N-formyl-Met-Leu-Phe (fMLF)-activated human neutrophils. Human neutrophils were pre-incubated with dimethylsulfoxide (DMSO), (**A**) PFE (3 and 10 μg/ml), or (**B**) PP2 (SFK inhibitor, 1 μM) before stimulation with fMLF (0.1 μM). All the Western blotting experiments were performed under the same condition. After transferring the blots onto nitrocellulose membranes, we immediately cropped the targeted blots according to referenced indicating markers, and then targeted proteins were immunoblotted with its specific monoclonal antibody. (**A,B**) Representative images from one of four independent experiments of Western blotting using anti-phospho antibodies directed against SFKs, Src, and Lyn were shown. Bands on the blots were analyzed using a densitometer, and the quantitative ratios for all samples were normalized to the corresponding total protein or to glyceraldehyde 3-phosphate dehydrogenase (GAPDH).

**Figure 4:**
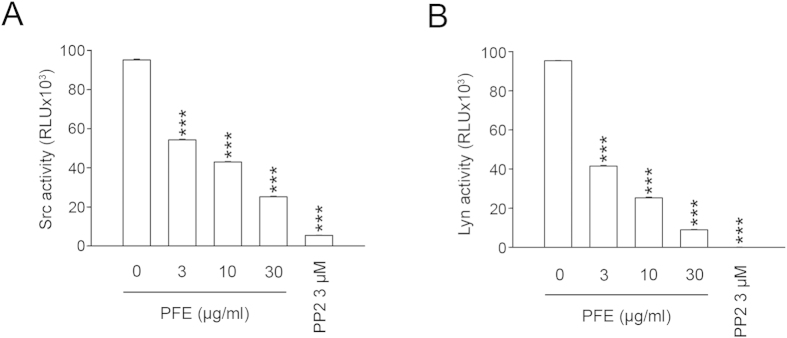
*P. frutescens* extract (PFE) inhibited Src and Lyn enzymatic activity. (**A**) Src (3 ng/ml) or (**B**) Lyn (3 ng/ml) was incubated with dimethylsulfoxide (DMSO, as control), PFE (3, 10, and 30 μg/ml), or PP2 (3 μM) for 10 min, and then ATP/substrate was added to the reaction mixture for 60 min prior to measurement of Src or Lyn activity, as described in the Methods section. Data are expressed as the mean ± standard error of the mean, n = 3, ***p < 0.001, as compared to the control group.

SFKs are protein-tyrosine kinases that play important roles in neutrophil activation triggered by the chemotactic peptide, fMLF^24^. Our immunoblotting analyses demonstrated that fMLF stimulated the phosphorylation of SFKs (Tyr416), Src (Tyr416), and Lyn (Tyr 396) in human neutrophils; PFE attenuated phosphorylation of these proteins (Fig. 3A). PP2, a selective inhibitor of SFKs, also inhibited phosphorylation of these SFKs (Fig. 3B). In addition, we investigated whether PFE inhibited the enzymatic activities of Src and Lyn. The results of this analysis indicated that PFE inhibited Src and Lyn tyrosine kinase activities in a concentration-dependent manner (IC_50_ = 5.21 ± 0.36 μg/ml and 2.51 ± 0.29, respectively) (Fig. 4A,B). PP2 was used as a positive control.

### PFE attenuated the phosphorylation of Burton’s tyrosine kinases (Btk)

**Figure 5:**
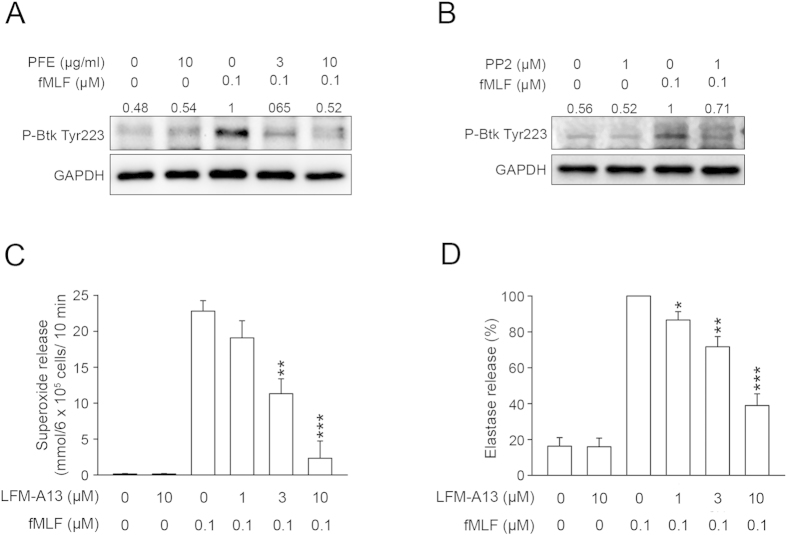
*P. frutescens* extract (PFE) and PP2 attenuated the phosphorylation of Bruton’s tyrosine kinase (Btk) in N-formyl-Met-Leu-Phe (fMLF)-activated human neutrophils. Human neutrophils were pre-incubated with dimethylsulfoxide(DMSO), (A) PFE (3 and 10 μg/ml), or (B) PP2 (1 μM) before stimulation with fMLF (0.1 μM). (**A,B**) Phosphorylation of Btk was detected by immunoblotting. Representative images from one of three independent experiments are shown. (**C**) O_2_˙^–^ production and (**D**) elastase release were measured spectrophotometrically in human neutrophils incubated with DMSO or a Btk inhibitor (LFM-A13; 1, 3, and 10 μM) and then activated with fMLF/cytochalasin B (CB). Data are expressed as the mean ± standard error of the mean, n = 4, *p < 0.05, **p < 0.01, ***p < 0.001, as compared to the fMLF group.

Btk belongs to the Tec family of tyrosine kinases, which includes Itk, Tec, Txk, and Bmx^25^,^26^,^27^. Btk is involved in G-protein coupled receptor (GPCR) signal transduction^27^,^28^,^29^. Immunoblotting for phospho-Btk demonstrated that both PFE (3 and 10 μg/ml) and PP2 (1 μM) attenuated the phosphorylation of Btk Tyr223 (Fig. 5A,B). Furthermore, we used a Btk inhibitor, LFM-A13, to explore the role of Btk in the regulation of neutrophil activation. Figure 5C,D revealed that LFM-A13 (1, 3, and 10 μM) decreased O_2_˙^–^ and elastase release from activated human neutrophils, with an IC_50_ of 2.50 ± 0.21 μM and 5.85 ± 1.36 μM, respectively.

### PFE reduced the phosphorylation of Vav

**Figure 6:**
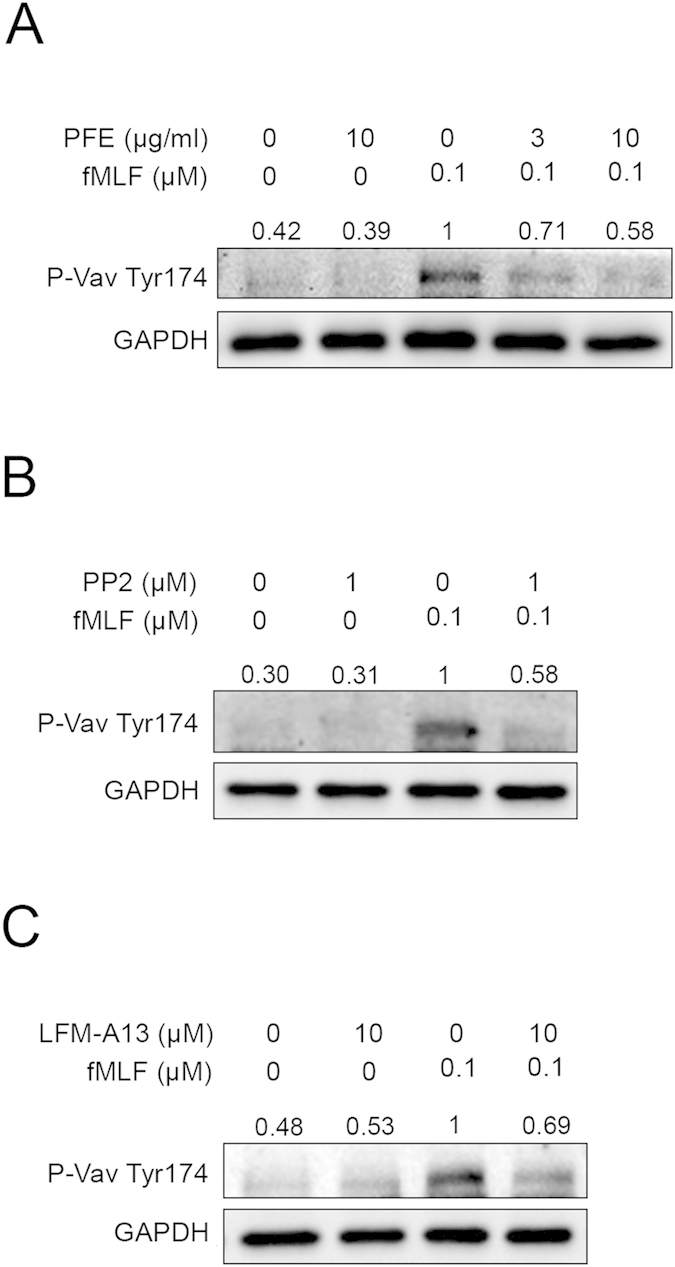
*P. frutescens* extract (PFE), PP2, and LFM-A13 attenuated the phosphorylation of Vav in N-formyl-Met-Leu-Phe (fMLF)-activated human neutrophils. Human neutrophils were incubated with dimethylsulfoxide (DMSO), (**A**) PFE (3 and 10 μg/ml), (**B**) PP2 (1 μM), or (**C**) LFM-A13 (10 μM) before stimulation with fMLF (0.1 μM). Phosphorylated Vav and glyceraldehyde 3-phosphate dehydrogenase (GAPDH) were detected by immunoblotting. Representative images from one of four independent experiments are shown.

As a member of the guanine nucleotide exchange factors (GEFs) family, Vav activates Rac activity by replacing the GDP in its switch protein with GTP, leading to the activation of NADPH oxidase^24^,^30^,^31^,^32^. Previous studies showed that SFKs induced Vav phosphorylation and activation^33^. Therefore, we explored whether Vav was involved in PFE-caused inhibition. Immunoblotting analyses revealed that PFE (3 and 10 μg/ml), PP2 (1 μM), and LFM-A13 (10 μM) inhibited the phosphorylation of Vav Tyr174 in fMLF-activated neutrophils (Fig. 6).

### PFE, but not PP2,decreased intracellular Ca^2+^ mobilization

**Figure 7:**
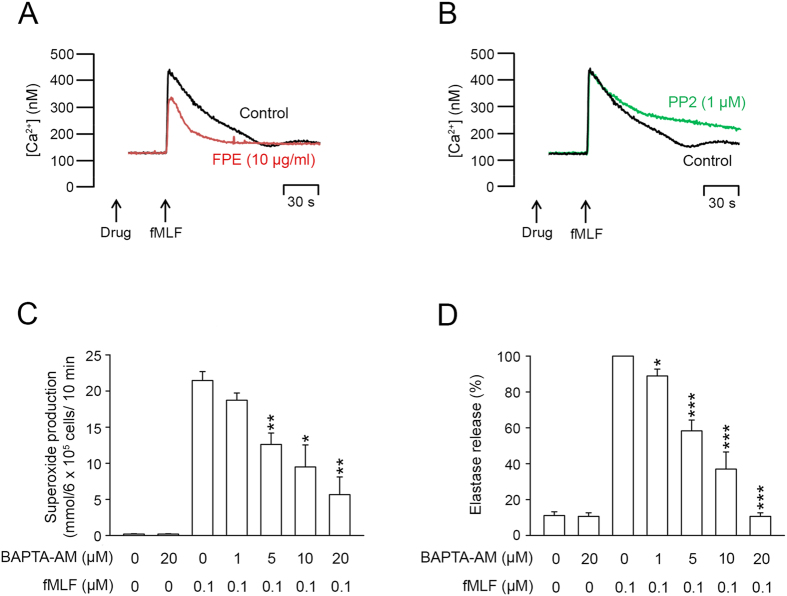
*P. frutescens* extract (PFE), but not PP2, reduced intracellular Ca^2+^ mobilization in N-formyl-Met-Leu-Phe (fMLF)-activated human neutrophils. Fluo-3/AM-labeled human neutrophils were incubated with (**A**) PFE (10 μg/ml) or (**B**) PP2 (1 μM) for 5 min before stimulation of fMLF (0.1 μM). Fluorescence was monitored in a spectrofluorometer (n = 3). (**C**) O_2_˙^−^ production and (**D**) elastase release were measured spectrophotometrically in fMLF-activated neutrophils incubated with dimethylsulfoxide (DMSO) or 1, 2-bis(o-aminophenoxy)ethane-N,N,N′,N′-tetraacetic acid tetrakis-acetoxymethyl ester (BAPTA-AM; 1-20 μM) and then activated with fMLF/cytochalasin B (CB). Data are expressed as the mean ± standard error of the mean, n = 4, *p < 0.05, **p < 0.01, ***p < 0.001, as compared to the fMLF group.

Ca^2+^ is an important second messenger in human neutrophil activations. Stimulation of neutrophils with fMLF elicited a rapid increase of the intracellular Ca^2+^ concentration ([Ca^2+^]_i_). Our results demonstrated that PFE (10 μg/ml) diminished the amplitude of this increase (Fig. 7A); while PP2 (1 μM) increased the time required for [Ca^2+^]_i_ to return to half of its peak value (*t*_1/2_, Fig. 7B). Moreover, we found that BAPTA/AM (1–20 μM), a membrane permeable Ca^2+^ chelator, reduced O_2_˙^–^ and elastase release from neutrophils activated by fMLF (Fig. 7C,D).

### Fingerprint chromatogram of PFE

**Figure 8:**
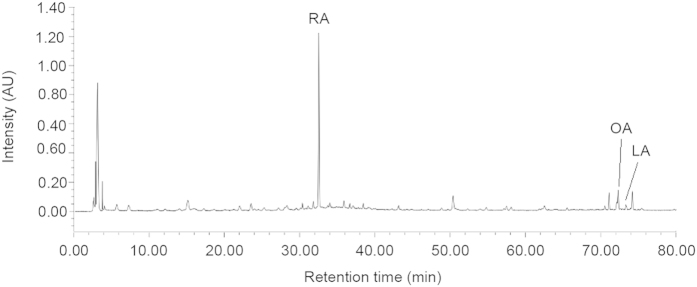
Chromatography analysis of *P. frutescens* extract (PFE). The concentration of PFE for HPLC analysis was 4 mg/ml. The injection volume was 20 μl, and the UV detection wavelength was set at 210 nm. The HPLC fingerprint showed peaks of rosmarinic acid (RA), oleanolic acid (OA), and linoleic acid (LA) at 32.5, 72.1, and 73.4 min, respectively.

The fingerprint chromatogram of PFE was obtained by high performance liquid chromatography (HPLC) for quality control. The detector wavelength at 210 nm showed superior separation compared with other wavelengths. Identification of PFE was dependent on retention time and UV spectra in comparison with the standard. The fingerprint chromatogram showed peaks of rosmarinic acid, oleanolic acid, and linoleic acid eluted at 32.5, 72.1, and 73.4 min, respectively (Fig. 8).

## Discussion

*P. frutescens* is a popular vegetable and food condiment that also commonly utilized as an herbal medicine in many Asian countries. Previous studies demonstrated that *P. frutescens* has important anti-inflammatory effects^5^,^6^. Nevertheless, little is known about the pharmacological mechanisms underlying these effects in human neutrophils. In the present study, we found that PFE inhibited the respiratory burst, degranulation, and chemotactic migration of fMLF-activated human neutrophils through inhibiting SFKs (Src and Lyn) pathway and intracellular Ca^2+^mobilization.

Many studies have revealed that compounds found in *P. frutescens* have anti-oxidative effects^7^,^8^. In this study, we found that PFE reduced O_2_˙^–^ production and ROS formation in fMLF-induced human neutrophils. We also used a cell-free xanthine/xanthine oxidase system and found that higher concentration of PFE (10 μg/ml and 30 μg/ml) had direct O_2_˙^–^ scavenging activity (IC_50_ = 23.43 ± 0.34 μg/ml; data not shown); this finding was consistent with a previous report^7^. These findings suggested that PFE reduced ROS levels, either by modulating cellular signaling or by direct free radical scavenging activity.

SFKs are non-receptor tyrosine kinases that regulate cell growth, differentiation and activation via various intracellular signaling pathways. Several SFKs such as Lyn, Hck and Fgr are expressed in human neutrophils^34^,^35^. These kinases are involved in fMLF-activated signal transduction processes^24^,^36^,^37^,^38^. In our studies, a selective inhibitor of SFKs (PP2) significantly reduced O_2_˙^–^ production and elastase release in neutrophils stimulated with fMLF. CD11b expression and chemotactic migration of fMLF-induced neutrophils were also reduced in the presence of PP2 (data not shown). Other studies^24^ and our data support the role of SFKs in signal transduction triggered by fMLF receptors and neutrophil activation. Furthermore, we demonstrated that PFE inhibited the phosphorylation of the SFKs, Src, and Lyn in fMLF-activated neutrophils. Furthermore, we found that PFE directly inhibited the enzymatic activities of Src and Lyn. These data suggestedthat PFE influenced the activities of SFKs, which plays an important role in the functional responses to fMLF.

Btk belongs to the Tec family of tyrosine kinases. Previous reports have revealed a tight relationship between SFKs and the Tec family^27^,^29^,^39^. The phosphorylation of Btk at Tyr551and subsequent autophosphorylation at Tyr223, which is necessary for its full activation, are closely correlated with the activity of SFKs^40^,^41^. In the present study, we found that an inhibitor of Btk (LFM-A13) reduced O_2_˙^−^ production and elastase release in fMLF-activated human neutrophils, which was consistent with a previous study^29^ describing the role of Btk in neutrophil activation. Moreover, we observed that the phosphorylation of Btk was significantly inhibited by PP2, suggesting that Btk was downstream of SFKs. In addition, Vav acts as a GEF for Rac, which is a subfamily of the RHO family, leading to the activation of NADPH oxidase^24^,^30^,^31^,^32^. Fumagalli *et al*. demonstrated that SFKs phosphorylated Vav in the signaling pathway triggered by fMLF, resulting in neutrophil activation^24^. Our immunoblotting data showed that both PP2 and LFM-A13 inhibited the phosphorylation of Vav (Fig. 5C), suggesting that SFKs and Btk modulated Vav activation. In agreement with this finding, PFE significantly inhibited the fMLF-induced phosphorylation of Btk and Vav in human neutrophils. Taken together, the results of this study provided evidence for the role of SFKs/Btk/Vav in human neutrophil activation triggered by fMLF, and indicated that PFE regulated human neutrophil activation by inhibiting this SFKs/Btk/Vav signaling pathway.

Ca^2+^ is a vital intracellular second messenger that contributes to neutrophil activation^42^,^43^. The binding of fMLF to GPCR triggers a rapid and transient increase in [Ca^2+^]_i_, resulting in neutrophil activation. Some specific inhibitors of Ca^2+^ signaling have been investigated as anti-inflammatory drugs because of their suppressive effects on neutrophil functions^44^. Our experiments using a Ca^2+^ chelator (BAPTA-AM) proved that increased [Ca^2+^]_i_ was required for the fMLF-induced respiratory burst and elastase release. Furthermore, our study showed that PFE reduced the fMLF-induced amplification of [Ca^2+^]_i_. However, PP2 prolonged the time required for [Ca^2+^]_i_ to resume its original equilibrium concentration. These findings inferred that the Ca^2+^ mobilization inhibited by PFE was independent of SFKs. Intracellular Ca^2+^ transients and SFKs are both important signal transduction pathways in fMLF-induced neutrophils^23^. Obviously, additional work is required to define the signal events linking SFKs Ca^2+^ mobilization in human neutrophils.

**Figure 9:**
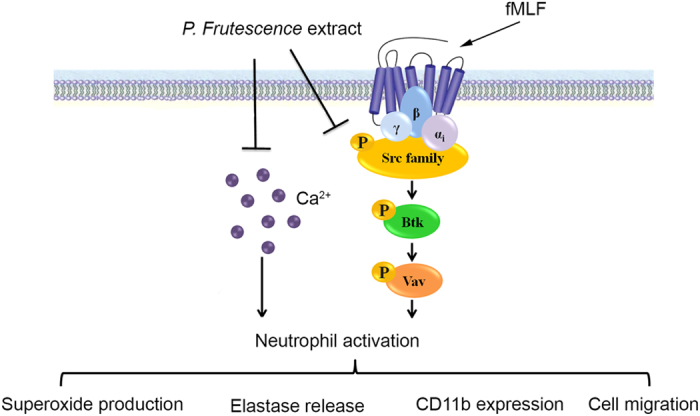
Schematic diagram illustrating the proposed mechanism of action of *P. frutescens* extract (PFE) in human neutrophils. PFE inhibits O_2_˙^–^ production, elastase release, reactive oxygen species (ROS) formation, CD11b expression, and chemotactic migration in N-formyl-Met-Leu-Phe (fMLF)-activated human neutrophils. PFE inhibits fMLF-induced human neutrophil activation by blocking Src family kinases (SFKs), Bruton’s tyrosine kinase (Btk), and Vav, in addition to reducing Ca^2+^ mobilization.

Based on these findings, we conclude that PFE significantly inhibited fMLF-induced human neutrophil activation, including O_2_˙^–^ production, elastase release, ROS formation, CD11b expression, and chemotactic migration. PFE inhibited activation of SFKs and Ca^2+^ mobilization, which represent two signaling pathways involved in fMLF-induced neutrophil activation (Fig. 9). Because the PFE used in the present study was a crude extract of *P. frutescens*, any of the chemical components of PFE may be involved in the effects and mechanisms described above. Further studies aimed at clarifying whether these two signaling pathways are regulated by one or more of the different chemical constituents of PFE should contribute to the development of more effective therapeutic options.

## Methods

### Preparation of *P. frutescens* extract

PFE was prepared by our co-author, Dr. Leu. *P. frutescens* (L.) Britt leaf powder was purchased from the Sun-Ten Pharmaceutical Co., Ltd. (Taipei, Taiwan). This contained the *P. frutescens* extract (67%) and corn starch (33%). The powder (1 g) was suspended in 10 ml ethanol at 37 °C for 4 h and then centrifuged at 5000 *g* for 20 min. The supernatant was filtered and lyophilized. The resulting PFE was suspended in dimethylsulfoxide (DMSO) at a concentration of 10 mg/ml and stored at −20 °C until use. The voucher specimen (CGU-NP-PFE-327) was secured at the Graduate Institute of Natural Products, College of Medicine, Chang Gung University, Taoyuan, Taiwan .

### Reagents

Dihydrorhodamine 123 (DHR123) and fluo-3 acetomethoxyester (fluo-3/AM) were obtained from Molecular Probes (Eugene, OR, USA). 1, 2-Bis(o-aminophenoxy)ethane-N,N,N′,N′-tetraacetic acid tetrakis-acetoxymethyl ester (BAPTA-AM) was from Tocris Bioscience (Ellisville, MO, USA). Antibodies directed against phosphorylated SFKs (Tyr416), Lyn, and phosphorylated Btk (Tyr223) were purchased from CellSignaling (Beverly, MA, USA). An antibody against phospho-Src (Tyr416) was purchased from Millipore (Billerica, MA, USA). Antibodies against phospho-Lyn (Tyr396), phospho-Vav (Tyr174), and glyceraldehyde 3-phosphate dehydrogenase (GAPDH) were purchased from EnoGene (NY, USA). An antibody against Src was purchased from Epitomic (Burlingame, CA, USA). FITC-labeled anti-human CD11b was purchased from eBioscience (San Diego, CA, USA). Src and Lyn kinase enzyme systems were purchased from Promega (Madison, WI, USA). The Moxi Z automatic cell counter was purchased from ORFLO (Hailey, ID, USA). All other reagents were purchased from Sigma-Aldrich (St. Louis, MO, USA).

### Isolation of human neutrophils

This study protocol was investigated and approved by the Institutional Review Board at Chang Gung Memorial Hospital, and written informed consent was obtained from every volunteer. The methods were carried out in accordance with the approved guidelines. Blood was drawn from healthy volunteers (aged 20–30 years) who had no congenital or systemic disease and did not take any medicine during the week prior to sample collection. Human neutrophils were isolated using a standard method of dextran sedimentation prior to centrifugation in a Ficoll-Hypaque gradient and hypotonic lysis of red blood cells^45^. The granulocyte layer was harvested and resuspended in Ca^2+^-free Hank’s balanced salt solution (HBSS) at pH 7.4, and maintained at 4 °C until use. Greater than 98% cell viability was confirmed by trypan blue exclusion. The data presented for each specific experiment were derived from 4 to 7 samples.

### Measurement of O_2_˙^–^ production

The reduction of ferricytochrome *c* was used to measure O_2_˙^–^ release from human neutrophils^46^. Neutrophils (6 × 10^5^ cells/ml) were incubated with 0.5 mg/ml ferricytochrome *c* and 1 mM CaCl_2_ at 37 °C, and then treated with DMSO (as control), PFE, 4-amino-5-(4-chlorophenyl)-7-(*t*-butyl)pyrazolo[3,4-d]pyrimidine (PP2, a selective inhibitor of SFKs), or 2-cyano-*N*-(2,5-dibromophenyl)-3-hydroxy-2-butenamide (LFM-A13, a Btk inhibitor), or BAPTA-AM (a Ca^2+^ chelator) for 5 min. Neutrophils were activated by adding fMLF (0.1 μM) with cytochalasin B (1 μg/ml) pretreatment (fMLF/CB). The change in absorbance at 550 nm reflected the reduction of ferricytochrome *c* and was monitored continuously using a spectrophotometer (U-3010; Hitachi, Tokyo, Japan). O_2_˙^–^ release was calculated as described previously^47^.

### Assessment of elastase release

Human neutrophils (6 × 10^5^ cells/ml) were equilibrated with an elastase substrate (MeO-Suc-Ala-Ala-Pro-Val-p-nitroanilide, 100 μM) at 37 °C for 2 min and then incubated with DMSO, PFE, PP2, LFM-A13, or BAPTA/AM for 5 min. Cells were activated by fMLF (0.1 μM)/CB (0.5 μg/ml) for a further 10 min. Elastase release was determined by measuring the changes in absorbance at 405 nm using a spectrophotometer (U-3010; Hitachi, Tokyo, Japan). The results were expressed as a percentage of the elastase release in the fMLF/CB-activated, drug-free, control group^48^.

### Determination of ROS formation

Cell-permeable DHR123, which is not fluorescent until oxidized, was used to detect intracellular ROS. Human neutrophils (1 × 10^6^cells/ml) were incubated in HBSS containing DHR123 (2 μM) for 10 min at 37 °C. They were then treated with PFE or PP2 for 5 min, and then activated by fMLF (0.1 μM)/CB (0.5 μg/ml) for 15 min. The change in fluorescence was analyzed by flow cytometry (FACSCalibur™; BD Bioscience, San Jose, CA, USA).

### Evaluation of LDH release

LDH release was used as an indicator of cell membrane integrity and served as a general means to assess cytotoxicity. We used commercially available reagents (Promega) to determine the LDH level. Human neutrophils were treated with PFE (10 μM) for 60 min. LDH assay reagents was then added to the supernatant. LDH release was expressed as a percentage of the amount of enzyme liberated following incubation of human neutrophils with 0.1% Triton X-100 for 30 min at 37 °C.

### Measurement of CD11b expression

CD11b/CD18 is a heterodimeric glycoprotein that is expressed on the plasma membrane of neutrophils. Neutrophils (5 × 10^6^ cells/ml) were preincubated with PFE, PP2, or LMF-A13 for 5 min and then activated by fMLF (0.1 μM)/CB (0.5 μg/ml) for a further 5 min. The reaction was stopped by placing the cells on ice prior to centrifugation at 4 °C. The supernatant was discarded and the cells were resuspended in 0.5% bovine serum albumin for staining using a FITC-labeled-anti-CD11b antibody (1 μg) for 90 min at 4 °C. The fluorescence intensity of FITC-labeled anti-CD11b was then monitored using flow cytometry.

### Chemotactic migration assay

Human neutrophil chemotactic migration was assessed using a microchemotaxis chamber with a 3-μm filters (Millipore)^49^. Neutrophils (5 × 10^6^ cells/ml) were incubated with DMSO or PFE (3 and 10 μg/ml) for 5 min at 37 °C and then placed into the top chamber. HBSS containing fMLF (0.1 μM) was added to the bottom chamber. After incubation in a 5% CO_2_ incubator for 60 min, the number of cells that migrated was determined by a MoxiZ automatic cell counter (ORFLO).

### Immunoblotting

The neutrophils were treated with DMSO, PFE (3 and 10 μg/ml), PP2 (1 μM), or LFM-A13 (10 μM) for 5 min before stimulation with or without fMLF (0.1 μM) for another 30 sec at 37 °C. Cells were lysed in a buffer containing 50 mM HEPES (pH 7.4), 100 mM NaCl, 1 mM EDTA, 2 mM Na_3_VO_4_, 10 mM p-nitrophenyl phosphate, 5% β-mercaptoethanol, 1 mM phenylmethanesulfonyl fluoride, 1% protease inhibitor cocktail (Sigma-Aldrich), and 1% Triton X-100. After brief sonication, the samples were centrifuged at 14,000 rpm for 20 min at 4 °C to yield whole-cell lysates. The proteins were then subjected to sodium dodecyl sulfate-polyacrylamide gel electrophoresis and transferred to membranes. After being blocked with 5% nonfat milk in a mixture of Tris-buffered saline and Tween 20 (TBS-T), the membranes were incubated with diluted primary antibodies at 4 °C overnight;these were anti-phospho-Src family (Tyr416), anti-phospho-Src (Tyr416), anti-Src, anti-phospho-Lyn (Tyr396), anti-Lyn, anti-phospho-Btk (Tyr223), anti-phospho-Vav (Tyr174), and anti-GAPDH antibodies. The membranes were washed with 0.05% TBS-T and incubated with diluted horseradish peroxidase-conjugated secondary antibodies at room temperature for 1 h. After being washed, enhanced chemiluminescence solution was added to the membranes and protein bands were detected using the BioSpectrum^®^ Imaging System (UVP; Upland, CA, USA).

### Src and Lyn kinase activity

Src and Lyn kinase activity were determined by the ADP-Glo^TM^ kinase assay kit (Promega) according to the manufacturer’s instructions. Briefly, Src or Lyn kinase reactions were performed using a buffer containing Src or Lyn kinase, Src or Lyn kinase substrate, ATP (50 μM), and PFE (3, 10, and 30 μg/ml) or PP2 (3 μM) for 60 min. ADP-GloTM reagent was added to terminate the kinase reaction and deplete the remaining ATP. The luciferase/luciferin luminescence was recorded with a microplate reader (Infinite 200 Pro; Tecan, Männedorf, Switzerland).

### Examination of ([Ca^2+^]_i_)

Human neutrophils (6 × 10^5^ cells/ml) were incubated with Fura-3/AM (2 μM) at 37 °C for 30 min, followed by centrifugation and resuspension in HBSS solution containing CaCl_2_ (1 mM). The Fura-3/AM-labeled neutrophils were treated with PFE (10 μg/ml) or PP2 (1 μM) for 5 min. The [Ca^2+^]_i_ in response to fMLF (0.1 μM) was measured under continuous stirring using a spectrophotometer with an excitation wave length of 488 nm and an emission wavelength of 520 nm. Maximum and minimum fluorescence values were obtained by adding 0.05% Triton X-100 and 20 mM ethylene glycol tetraacetic acid (EGTA) sequentially at the end of each experiment.

### Fingerprint chromatogram of PFE

The HPLC analysis of PFE was conducted on a Waters HPLC system. The concentration of PFE was 4 mg/ml. The separation was performed using a reverse-phase column (Cosmosil 5C18-MS-II, 5 μm, 4.6 mm ×250 mm I.D.) connected with a guard column (Lichrospher RP-18 end-capped, 5 μm, 4.0 mm ×10 mm I.D.). The elution flow rate was 1.0 ml/min with a mobile phase gradient of A-B (A = 0.085% H_3_PO_4_, B = CH_3_CN), which was varied as follows: 0–10 min, 90–85% A, 10–15% B; 10–20 min, 85–80% A, 15–20% B; 20–30 min, 80–60% A, 20–40% B; 30–55 min, 60–35% A, 40–55% B; 55–65 min, 35–0% A, 65–100% B; 65–80 min, 0–90% A, 100–10% B. The injection volume was 20 μl, and the UV detection wavelength was set at 210 nm.

### Statistical analysis

Results were expressed as mean ± standard error of the mean (SEM). Computation of the 50% inhibitory concentration (IC_50_) was computer-assisted (PHARM/PCS v.4.2). Statistical comparisons were made between groups using Student’s *t*-test. Values of *p* less than 0.05 were considered to be statistically significant.

## Additional Information

**How to cite this article**: Chen, C.-Y. *et al*. Anti-inflammatory effects of *Perilla frutescens* in activated human neutrophils through two independent pathways: Src family kinases and Calcium. *Sci. Rep*. **5**, 18204; doi: 10.1038/srep18204 (2015).
